# Oral Habits during the Lockdown from the SARS-CoV-2 Pandemic in the Romanian Population

**DOI:** 10.3390/medicina58030387

**Published:** 2022-03-05

**Authors:** Mariana Cărămidă, Mihaela Adina Dumitrache, Ana Maria Cristina Țâncu, Roxana Romanița Ilici, Radu Ilinca, Ruxandra Sfeatcu

**Affiliations:** 1 Oral Health and Community Dentistry Department, Faculty of Dental Medicine, “Carol Davila” University of Medicine and Pharmacy, 17-21 Calea Plevnei Street, Sector 1, 010221 Bucharest, Romania; mariana.caramida@umfcd.ro (M.C.); mihaela.dumitrache@umfcd.ro (M.A.D.); ruxandra.sfeatcu@umfcd.ro (R.S.); 2 Complete Denture Department, Faculty of Dental Medicine, “Carol Davila” University of Medicine and Pharmacy, 17-21 Calea Plevnei Street, Sector 1, 010221 Bucharest, Romania; 3 Prosthesis Technology and Dental Materials Department, Faculty of Dental Medicine, “Carol Davila” University of Medicine and Pharmacy, 17-21 Calea Plevnei Street, Sector 1, 010221 Bucharest, Romania; roxana.ilici@umfcd.ro; 4 Biophysics Department, Faculty of Dental Medicine, “Carol Davila” University of Medicine and Pharmacy, 17-21 Calea Plevnei Street, Sector 1, 010221 Bucharest, Romania

**Keywords:** COVID-19, SARS-CoV-2, oral habits, dental hygiene, smoking behavior, eating patterns

## Abstract

*Background and Objectives*: The SARS-CoV-2 pandemic led to changes in population daily patterns. In order to adapt oral health promotion measures for future similar conditions, the main objective of the study was to assess changes in dental hygiene and eating and smoking habits during the government lockdown in Romania. *Materials and Methods*: The cross-sectional study was conducted immediately after the end of the lockdown and consisted of 800 adult subjects. Data collection was done via an online survey. Participants were divided into two groups: non-medical/dental practitioners (N-M/D group) and medical/dental practitioners (M/D group). *Results*: An increased use of dental floss from 27% (pre-lockdown) to 30.5% (during lockdown) was identified in the M/D group, while the manual toothbrush usage increased to 64.8% (during lockdown) from 61.7% (pre-lockdown) in the N-MD/group. No significant differences regarding toothbrushing frequency were observed in either group. A change in the number of daily snacks was identified in both groups (3–4 snacks per day: from 11% to 20.2% in the N-M/D group, from 13.1% to 22.2% in the M/D group). The consumption of sweets as a preferred snack was also noticed. A decrease of tobacco consumers was assessed in the lockdown period (from 66.6% to 60.4% in the M/D group, from 68.5% to 61.9% in the N-/M/D group). *Conclusions:* Oral habits were changed during the pandemic lockdown through the increase in the frequency of the consumption of snacks and sweets and the decrease in frequency of smokers. Only minor changes were observed in oral hygiene.

## 1. Introduction

Oral health, in general, is impacted by multiple categories of risk factors, but in the particular case of dental caries and periodontal disease, a significant role is played by behavioral risk factors [1,2]. These are related to people’s lifestyle and choices over time, and oral hygiene habits, diet and smoking status are those behaviors that are both significant and controllable through education and behavioral change [1,2,3].

The year 2020 was marked by the global spread of SARS-CoV-2 virus, responsible for the severe acute respiratory syndrome, which is highly contagious and with great risks for overall health; thus, the World Health Organization declared a pandemic in March 2020 [4,5].

As in many counties, in Romania the state of emergency was established on March 16 and, starting with the last week of March until 15 May, a lockdown was implemented. During that quarantine (25 March–15 May 2020) the restrictions forced individuals to change their routine and a part of these restrictions impacted, indirectly, oral health-related behaviors; namely, educational activity in schools and universities were temporary interrupted, in most working fields employers switched to working from home or they stopped working and individuals were allowed to leave their houses only for certain essential reasons and on a strict schedule, limiting the access to oral health care services only for dental emergencies.

Oral health care services were negatively influenced, both because the activity in the dental clinics were forced to be reorganized in order to limit the spread of the virus [6,7,8,9,10,11], but also because a great part of the dentists and periodontists were under significant psychological pressure and distress levels [12,13,14]. Months after reopening the dental offices, studies showed that the probability of COVID-19 contamination within dental clinics was very low [15]. On the other hand, dental patients were underserved both because they were forced to interrupt their treatments during the lockdown and to access only dental emergency treatments for acute issues and they had to postpone their dental treatment [16,17,18]. Moreover, some of them were afraid to attend the dental check-ups because of their fear for COVID-19 contamination [16]; therefore, they cancelled the regular dental visits, thus increasing the risk for developing new oral health issues or relapse. In addition, recently published research [19] showed that periodontal disease, as chronic inflammatory disease, increases the risk for Intensive Care Unit attendance and even death among patients with COVID-19 infection, since any chronic inflammatory disease contributes to the amplification of complications and negative prognosis of this COVID-19-induced infection [20].

When it comes to prevention of oral diseases, there are some carious and periodontitis risk factors, such as smoking and a high sugar diet, that are related to lifestyle and that are common to general health issues [2]. A high intake of sugary food and beverages increases the risk for dental caries, besides the risk for obesity, cardio-vascular disease, diabetes and cancers [2,21]. In the COVID-19 context, the risk for severe complication of infections with SARS-CoV-2 were higher in people with these particular systemic diseases and conditions [22], which the Oral Health Organization reported even from the beginning of the pandemic, as well as among patients affected by untreated periodontal inflammation [19]. Thus, in ideal conditions, these aspects might have raised awareness in the general population so that people would change the behaviors in favor of a healthy lifestyle. On the other hand, the pandemic in general, and the lockdown in particular, had a major emotional impact on people [23], and under stressful conditions individuals tend to adopt unhealthy habits to help them cope [24,25]. When people spend a lot of time at home, in stress and boredom, two conditions experienced during the lockdown, they tend to increase the frequency of smoking [26,27] and meals/snacks and, in addition, of consuming “comfort food”, which in general is sugar-rich food [28,29,30,31]. Moreover, in regard to oral hygiene, time spent home during the lockdown offered people more time to perform and pay attention to routines beneficial for their oral health.

The COVID-19 pandemic has also affected the daily life routine and eating habits. Working from home, the routine of home delivery fast foods, coupled with delayed dental checkups, increased the risk dental diseases. The presence of caries is both an important indicator of oral health status and a condition that can lead to further complications with respect to more severe dental diseases. It is therefore preferable to diagnose caries as soon as possible in order to minimize the destructive effects they generate, and saliva tests results were found to be a relevant when it comes to management of dental routine checkups and prophylactic therapy [32]. Commercially available herbal mouthwashes, consumed in moderate amounts, were found to have positive effects on caries prevention, whereas excessive, long-term use of these mouthwashes was found to be responsible for mouth flora imbalance and teeth staining [33]. Caries occurrence is also promoted by use of acidic drinks by means of acid etiology. This risk factor is becoming more important since post-modern diets, especially when it comes to teenagers and young adults, include large amounts of sweetened carbonated soft drink consumption [34].

The study aimed to assess differences in oral hygiene routine as well as smoking and eating habits during the first lockdown in Romania induced by the COVID-19 pandemic.

## 2. Materials and Methods

### 2.1. Survey Methodology

The cross-sectional study was conducted in May 2020, in the last two weeks of the two-month lockdown that was implemented in Romania during the first wave of the COVID-19 pandemic. The present study was designed by the Department of Oral Health and Community Dentistry, Faculty of Dental Medicine of “Carol Davila” Medicine and Pharmacy University, Bucharest, Romania. The assessment of the changes in oral health-related behaviors was performed using a self-administered online questionnaire that was distributed and available for completion for two weeks. The estimated filling in time for the questionnaire was 5 min. The questionnaire was distributed via digital platforms commonly used for communication (e-mail, instant messaging, social media) and individuals who were invited to complete the form were, in advance, informed about the survey, in respect to the Declaration of Helsinki and the current European privacy law, pointing out the scientific aim of study, that the questionnaire was anonymous and that they had the right to interrupt the completion of the form at any moment.

### 2.2. Survey Population

Individuals invited to participate to the study were from general population, including professionals from medical and dental field. The exclusion criteria were children and adolescents younger than 18 years old. Because one of the hypotheses was that the behavioral patterns regarding oral health were different for subjects involved in the medical or dental sector, the sample was split in two subgroups by the occupational field: medical/dental group (M/D group) and non-medical/dental group (N-M/D group). The other hypothesis was that the oral hygiene routine was improved and that smoking and eating habits were impaired during lockdown, and that the evolution was different between the two subgroups.

### 2.3. Survey Questionnaire

The online questionnaire was formed by 17 items (open-ended and single/multiple-choice questions) distributed into two main sections: (1) oral hygiene, smoking and eating habits before and during the lockdown; and (2) socio-demographic data.

In the oral health-related behavior section, all questions and answers were formulated so that participants could select the particularities of their behaviors before and during the lockdown for the same aspects assessed. There were six questions regarding oral hygiene habits (frequency of toothbrushing, duration of toothbrushing, type of toothbrush used, additional oral hygiene products), three questions regarding eating habits (frequency of daily snacks between meals, type of food consumed as snacks, frequency of beverages consumption) and one question regarding smoking (smoking status/frequency). In addition, in this section we added one question to assess the self-perceived oral health status before the lockdown. In the socio-demographic section, data collected referred to age, sex, professional/educational field and work regimen during the lockdown (Table S1).

### 2.4. Statistical Analyses

Statistical analysis was performed using IBM SPSS Statistics v19 version (IMB Corp., Armonk, NY, USA). Descriptive statistics were used for frequencies and means for all of the categorical variables related to behaviors and demographic data, while for comparative analysis, non-parametric tests were used, including the Mann–Whitney U Test for inter-group comparison (between the two independent samples: medical/dental group and non-medical/dental group) and the Wilcoxon Signed Rank Test for intra-group comparison (between the before and during the pandemic, within each subgroup). For tests made within related samples, the Marginal Homogeneity Test was used for comparing categorical variables with more than two categories and the McNemar Test for comparing categorical variables with two categories (Yes/No). Post hoc McNemar Tests with Bonferroni correction were made when the Marginal Homogeneity Test proved to be statistically significant. Statistically significant differences were considered at the value of *p* < 0.05 and as highly statistically significant at *p* < 0.001.

## 3. Results

### 3.1. Participants

The study was conducted on a sample of 800 subjects with a mean age of 30.8 ± 11.58 years (range 18–75 years), with 649 (81.1%) women and 419 (52.4%) studying or working in the medical/dental field (M/D), while the others were active in other fields or with no professional/educational activity before the pandemic (N-M/D).

Among the subjects enrolled in the study, 43.9% (351) of subjects worked/studied from home during the lockdown, 42.1% (337) completely interrupted their professional activity during lockdown, 6.3% (50) of subjects continued their activity at their working place and 7.8% (62) of subjects worked both from home and at their working place.

Comparing the oral health-related behavioral patterns before the lockdown between the N-M/D group and M/D group, the results showed that there were statistically significant differences (*p* < 0.001) regarding oral hygiene habits and self-assessed oral health status, in favor of the medical/dental group, but no statistically significant differences regarding smoking and eating habits were found between the two groups (*p* > 0.05).

### 3.2. Oral Hygiene Habits

The frequency of toothbrushing did not change at a statistically significant level within either of the groups. However, the time spent for toothbrushing increased statistically significant in M/D group during the lockdown, with more subjects brushing for at least 2 min compared to pre-lockdown (*p* = 0.028). The Marginal Homogeneity Test showed significant differences in the N-M/D group (*p* = 0.024), but post hoc tests were not statistically significant; therefore, no significant differences were observed in the N-M/D group (Table 1).

On the other hand, in the N-M/D group, we found a statistically significant difference that more participants used manual toothbrushes during the lockdown (*p* = 0.036), and less participants used both manual and powered toothbrushes (*p* = 0.006). The Marginal Homogeneity Test also showed significant differences in the M/D group (*p* = 0.049), but post hoc tests were not statistically significant; therefore, no significant differences were observed in the M/D group (Table 1).

The use of additional hygiene products, such as dental floss, mouth rinses, interdental brushes, oral irrigators and toothpicks, was similar pre- and during the lockdown in both groups, except for dental floss in the M/D group, for which the frequency of daily users increased statistically significant from 27% pre-lockdown to 30.5% during lockdown (*p* = 0.012) (Table 1).

### 3.3. Eating Habits

The frequency of snacks between meals during the day increased and was highly statistically significant during the lockdown, with less participants declaring having no snacks at all during lockdown (11.3% in N-M/D group, 10.3% in M/D group) compared to pre-lockdown (16% in N-M/D group, 17.4% in M-D group), less participants declaring having 1–2 snacks per day during lockdown (64.3% in N-M/D group) compared to pre-lockdown (72.2% in N-M/D group) and almost twice as many participants declaring having 3–4 snacks per day (20.2% in N-M/D group, 22.2% in M/D group) compared to pre-lockdown (11% in N-M/D group, 13.1% in M/D group), or even >4 times per day in the N-M/D group (4.2% during lockdown vs. 0.8% pre-lockdown) (Table 2).

Regarding the type of food preferred for snacks, results showed that sweets were included in the daily snacks by statistically significant more participants in both groups during the lockdown (45.4% in N-M/D group, 52% in M/D group) than before (38.1% in N-M/D group, 44.6% in M/D group).

Other statistically significant differences were observed in M/D group in regard to the consumption of fruits, which increased during the lockdown (51.3% vs. 43.4%), and sandwiches, which decreased (6.4% vs. 10.3%), while in the N-M/D group, the increase was seen in the consumption of chips/popcorn (14.7% vs. 11.3%) and dairy products (14.4% vs. 9.7%) (Table 2).

In terms of beverage (non-alcoholic) consumption, only for the N-M/D group were significant differences detected based on the Marginal Homogeneity Test (*p* = 0.031), but post hoc tests were not significant; therefore, in both groups, the frequency of beverage consumption was not statistically different between the two times (during lockdown vs. pre-lockdown) (Table 2).

### 3.4. Smoking Habits

There was a statistically significant reduction of smokers during the lockdown seen in both groups of participants: there was a greater increase in number of participants who decided to not smoke at all during lockdown (68.5% in N-M/D group, 66.6% in M/D group) compared to pre-lockdown (61.9% in N-M/D group, 60.4% in M/D group); participants in the M/D group that smoked occasionally smoked significantly less during lockdown than pre-lockdown (8.6% vs. 12.6%), and participants in the N-M/D group that smoked 1–10 cigarettes/day smoked significantly less during lockdown than pre-lockdown (11.8% vs. 16%) (Table 3).

## 4. Discussion

In the present study, changes regarding the habits related to oral hygiene showed that, on the one hand, more subjects from the non-medical/dental group preferred the manual toothbrushes during the lockdown compared to pre-lockdown. On the other hand, more subjects from the medical/dental group used daily dental floss and exceeded the recommended duration of 2 min for toothbrushing during the lockdown compared to pre-lockdown period. When it comes to the habits related to eating, an increase in the frequency of daily snacking and in the consumption of sweets as the predominant product for snacking was observed during the lockdown compared to pre-lockdown, irrespective of the studied subgroup. Changes regarding smoking habits showed that the number of non-smokers increased during the lockdown compared to pre-lockdown, irrespective of the studied group. There was observed a decrease in occasional smokers in the medical/dental professionals subgroup, and in the daily smokers subgroup of a maximum of 10 cigarettes per day in the non-medical/dental group.

To the date when the present paper was written, other published results from similar research on adults in other populations were not found. However, research conducted on a group of Chinese children, assessed with regard to changes in oral hygiene routine induced by the quarantine, showed that 85% of them did not change the frequency of toothbrushing and, among those who did, the proportion was similar between those who increase and those who decreased the number of daily toothbrushing [35].

On the other hand, when it comes to eating behavior, it is well known that a diet rich in fresh fruits and dairy (low fat or no fat) and low in processed/sugary food or beverages has a significant positive impact not only on general health, but also oral health [36]. However, while eating is important for biological functioning, it is well-known that eating choices and behaviors are influenced by emotional state and cultural context. Thus, overeating of eating sugary or salty food might be compulsory in times of emotional stress like in this particular situation of the COVID-19 pandemic, and it is also a hard to change behavior [21]. In previous research conducted in Romania on a group of adolescents who received oral health-related education through experience learning for 2 years, the results show that the eating habit changes were at a lower level than other behaviors and often the changes were only temporary [37]. That is in accordance with another recent research conducted in the Netherlands that showed that diet is hard to change even with educational intervention [21].

Frequency of eating during the day increased in our study, as seen in the Polish population [38] during the COVID-19 lockdown period, but while Romanians declared having three snacks per day, most Polish declared having one or two snacks per day. In a similar study in United Arab Emirates, the percent of participants who were consuming at least five meals per day increased from 2.1% to 7% during the lockdown period [39]. In a United Kingdom study, 53% of participants declared an increase in the number of snacks during the lockdown period and the factors associated with overeating during the lockdown were observed with the following: a young age, feminine gender, lower education, negative mental health, suspicion or confirmation of COVID-19 infection, negative mental health experiences during pandemic and higher body mass index [40]. In the Netherlands, only one-tenth of the assessed participants in a similar study mentioned eating more often during the quarantine than before, and those who reported consuming more unhealthy food during the pandemic than before mentioned the following as reasons for their eating behavior: boredom, temptation of unhealthy products at home and more leisure time available [36].

Sweets were consumed daily by around half of participants in our study, similar to the proportion found in UAE population during COVID-19 quarantine [34]. In a multicenter study that assessed eating habit changes during the quarantine among adolescents from Spain, Italy, Brazil, Columbia and Chile, the daily intake of sweets increased from 14% before lockdown to 20% during the lockdown [41].

In our study, the reduction in consumption of non-alcoholic beverages, as seen also in Italian population [42], was noticed only among medical/dental professionals, and not for general population, while in the UAE population, daily consumption of beverages was seen in one-quarter of participants [39], while in our study population the proportion was less than one-fifth. No differences among adolescents from different countries were found in regard to beverage consumption during the pandemic in a multicenter study [41]. In the Netherlands population, the increase in purchasing beverages was observed among people with a middle education level [36].

The increase in fresh fruit consumption observed in our study was also seen in the Italian population [42], but in our study, the increase was observed only among medical/dental professionals. Compared to Polish population where two-thirds used to consume fresh fruits daily [38], only half of Romanians reported daily fruit consumption as a snack between meals, similar to the reports from the UAE population where half of the subjects consumed fruits at least once a day [39]. In the multicenter study on adolescents, the results showed an increase in fruit consumption (from one-quarter before the pandemic to one-third during the pandemic of at least one fruit per day) [42].

Globally, more than 1.4 billion people smoke, despite the well-known negative effects that their habit has both on general health [43] as well as on oral health, with an increased risk for periodontal inflammation and oral cancer [2]. In the present study, the decrease in frequency of smokers is in accordance to other studies in other European countries during pandemic [42], even though in our study, one-third of participants were smokers before the pandemic, while in the Italian studied population, only one-quarter smoke. In the Polish population, 45% of individuals reported more smoking during the lockdown [38]. Research conducted on UK population reported that two-thirds of the population increased smoking during the lockdown and the main reasons were the lack of professional activity, boredom and to relieve stress induced by the new and difficult COVID-19 pandemic, compared to other periods when pleasure or psychological addiction were more possible to be satisfied [44]. Therefore, psychological support is required for those who suffered from COVID-19 or from isolation [45].

Changes observed in the present study among medical and dental professionals, who have a healthcare educational and professional background, and thus are trained to be oriented to a healthy lifestyle, showed that under the exceptional circumstances of the COVID-19 pandemic, certain eating habits were changed unfavorably for oral health by the increased snacking and the consumption of sweets. In addition, the evolution of smoking habits was similar to that observed in the general population. Regarding the oral hygiene habits, improvements observed were limited to the use of dental floss. Furthermore, medical and dental practitioners, knowing the fact of being included among higher risk group during patient care, are exposed to increased psychological pressure and distress, along with effects of the pandemic [46,47].

However, the present survey was based on self-reported habits, which are associated with the limitation of possible underestimation or overestimation of the time spent or the frequency of certain habits, without the possibility for researchers to verify the real behaviors through objective assessment. Moreover, due to the limited time available for this research to be conducted amid the exceptional context of the first quarantine of the pandemic, the subjects enrolled might not have been fully representative for the Romanian general population. Therefore, for an accurate overview of the long-term impact of the behavioral change patterns reported in the present study on oral health status, there is a need to supplement these results with data from larger and representative population survey research along with clinical data from observational study on the same cohort, which is the aim of further research.

## 5. Conclusions

During the COVID-19 lockdown in Romania, changes regarding oral health-related habits were observed within the studied population. Oral hygiene behavior changed through an increased frequency of daily floss users as well as of subjects spending more than two minutes for toothbrushing among dental or medical professionals, and through an increase of users of manual toothbrushes among subjects from the general population, compared to pre-lockdown. Eating habits were changed by the increased frequency of snacks and of individuals preferring sweets as a snack, irrespective of their professional field. The frequency of smokers decreased during lockdown regardless the professional background of participants. The results uncover various changes in oral health habits during the COVID-19 pandemic and highlight the role that should be played in this regard by dental professionals and public health practitioners.

## Figures and Tables

**Table 1:** Oral hygiene changes between pre-lockdown and during the COVID-19 lockdown, among medical/dental professionals and the general population.

		N-M/D Group			M/D Group		
		During Lockdown % (*N)*	Before Lockdown % (*N*)	*p*-Value	During Lockdown % (*N*)	Before Lockdown % (*N*)	*p*-Value
Toothbrushing frequency				0.383 **			0.424 **
	>2 times/day	15% (57)	11.3% (43)		18.1% (76)	12.9% (54)	
	2 times/day	61.4% (234)	69.3% (264)		70.4% (295)	78.3% (328)	
	Once/day	21% (80)	18.4% (70)		10.3% (43)	8.4% (35)	
	Few times/week	2.4% (9)	0.8% (3)		1.2% (5)	0.5% (2)	
	Not at all	0.3% (1)	0.3% (1)		0% (0)	0% (0)	
Toothbrushing duration				0.024 *^,^**			<0.001 *^,^**
	30 s	8.9% (34)	9.2% (35)	1.000 ***	3.1% (13)	4.5% (19)	0.280 ***
	1 min	27.8% (106)	30.4% (116)	0.444 ***	14.3% (60)	17.2% (72)	0.236 ***
	2 min	40.7% (155)	39.1% (149)	1.000 ***	44.6% (187)	45.1% (189)	1.000 ***
	>2 min	22.6% (86)	21.3% (81)	1.000 ***	37.9% (159)	33.2% (139)	0.028 *^,^***
Type of toothbrush				0.001 *^,^**			0.049 *^,^**
	Manual	64.8% (247)	61.7% (235)	0.036 *^,^***	44.6% (187)	42.7% (179)	0.456 ***
	Powered	25.7% (98)	25.5% (97)	1.000 ***	34.1% (143)	34.1% (143)	1.000 ***
	Both manual and powered	9.4% (36)	12.9% (49)	0.006 *^,^***	21.2% (89)	23.2% (97)	0.345 ***
Additional oral hygiene products use	Dental floss	43.8% (167)	45.4% (173)	0.286 ^§^	69.9% (293)	71.4% (299)	0.405 ^§^
	Daily use of dental floss	15.5% (59)	15.5% (59)	1.000 ^§^	30.5% (128)	27% (113)	0.012 *^,§^
	Mouthwash	48.8% (186)	50.9% (194)	0.216 ^§^	72.1% (302)	72.6% (304)	0.839 ^§^
	Toothpicks	29.7% (113)	28.9% (110)	0.549 ^§^	7.4% (31)	7.4% (31)	1.000 ^§^
	Interdental brushes	11% (42)	11.8% (45)	0.453 ^§^	18.4% (77)	19.1% (80)	0.629 ^§^
	Oral irrigator	9.4% (36)	9.4% (36)	1.000 ^§^	16% (67)	15.3% (64)	0.664 ^§^

* Statistically significant, ** Related-Samples Marginal Homogeneity Test, *** Related-Samples McNemar Test with Bonferroni correction, ^§^ Related-Samples McNemar Test without Bonferroni correction. Abbreviations: N-M/D Group, non-medical/dental group; M/D Group, medical/dental group.

**Table 2:** Eating habits changes between pre-lockdown and during the COVID-19 lockdown, among medical/dental professionals and the general population.

		N-M/D Group			M/D Group		
		During Lockdown % (*N*)	Before Lockdown % (*N*)	*p*-Value	During Lockdown % (*N*)	Before Lockdown % (*N*)	*p*-Value
Snacks frequency				<0.001 *^,^**			<0.001 *^,^**
	Not at all	11.3% (43)	16% (61)	0.048 *^,^***	10.3% (43)	17.4% (73)	0.008 *^,^***
	1–2 times/day	64.3% (245)	72.2% (275)	0.024 *^,^***	64% (268)	66.3% (278)	1.000 ***
	3–4 times/day	20.2% (77)	11% (42)	<0.001 *^,^***	22.2% (93)	13.1% (55)	<0.001 *^,^***
	>4 times/day	4.2% (16)	0.8% (3)	<0.001 *^,^***	3.6% (15)	3.1% (13)	1.000 ***
Predominant food products consumed as snacks	Sweets	45.4% (173)	38.1% (145)	0.001 ^§^	52% (218)	44.6% (187)	0.003 ^§^
	Fruits	50.4% (192)	47.8% (182)	0.212 ^§^	51.3% (215)	43.4% (182)	0.002 ^§^
	Starchy food	28.6% (109)	25.7% (98)	0.118 ^§^	27.2% (114)	31% (130)	0.110 ^§^
	Sandwich	5.2% (20)	5.2% (20)	1.000 ^§^	6.4% (27)	10.3% (43)	0.021 ^§^
	Dairy products	14.4% (55)	9.7% (37)	0.003 ^§^	14.6% (61)	12.4% (52)	0.243 ^§^
	Chips/popcorn	14.7% (56)	11.3% (43)	0.037 ^§^	16.5% (69)	14.1% (59)	0.221 ^§^
	Various (a wide range of food products)	22.3% (85)	23.6% (90)	0.359 ^§^	18.9% (79)	18.6% (78)	1.000 ^§^
Non-alcoholic beverage consumption				0.031 *^,^**			0.753 **
	Not at all	19.2% (73)	18.1% (69)	1.000 ***	24.1% (101)	21% (88)	-
	Rarely	48% (183)	51.2% (195)	1.000 ***	42.7% (179)	48.2% (202)	-
	A few times a week	15.2% (58)	17.1% (65)	0.155 ***	16% (67)	15% (63)	-
	Once a day	9.2% (35)	6.6% (25)	1.000 ***	6.4% (27)	5.5% (23)	-
	A few times a day	8.4% (32)	7.1% (27)	0.525 ***	10.7% (45)	10.3% (43)	-

* Statistically significant, ** Related-Samples Marginal Homogeneity Test, *** Related-Samples McNemar Test with Bonferroni correction, ^§^ Related-Samples McNemar Test without Bonferroni correction. Abbreviations: N-M/D Group, non-medical/dental group; M/D Group, medical/dental group.

**Table 3:** Smoking habit changes between pre-lockdown and during the COVID-19 lockdown, among medical/dental professionals and the general population.

		N-M/D Group			M/D Group		
		During Lockdown % (*N*)	Before Lockdown % (*N*)	*p*-Value	During Lockdown % (*N*)	Before Lockdown % (*N*)	*p*-Value
Smoking frequency				0.006 *^,^**			0.002 *^,^**
	Not at all	68.5% (261)	61.9% (236)	<0.001 *^,^***	66.6% (279)	60.4% (253)	<0.001 *^,^***
	Occasionally	8.4% (32)	12.1% (46)	0.070 **	8.6% (36)	12.6% (53)	0.035 *^,^***
	1–10 cigarettes/day	11.8% (45)	16% (61)	0.040 *^,^***	12.9% (54)	13.8% (58)	1.000 ***
	11–20 cigarettes/day	9.2% (35)	8.4% (32)	1.000 ***	10% (42)	12.2% (51)	0.755 ***
	>20 cigarettes/day	2.1% (8)	1.6% (6)	1.000 ***	1.9% (8)	1% (4)	1.000 ***

* Statistically significant, ** Related-Samples Marginal Homogeneity Test, *** Related-Samples McNemar Test with Bonferroni correction. Abbreviations: N-M/D Group, non-medical/dental group; M/D Group, medical/dental group.

## Data Availability

The data presented in this study are available from the corresponding author upon reasonable request.

## Supplementary Materials

The following supporting information can be downloaded at: https://www.mdpi.com/article/10.3390/medicina58030387/s1, Table S1: Questionnaire on behavioral risk factors for oral health on quarantine during the COVID-19 pandemic.
