# Supplementary material for: Oral Habits during the Lockdown from the SARS-CoV-2 Pandemic in the Romanian Population
Source: Medicina (Kaunas). 2022 Mar 5;58(3):387. doi: 10.3390/medicina58030387 (PMC8950074; doi:10.3390/medicina58030387)
Supplement: Supplementary file 1 [file medicina-58-00387-s001.zip › medicina-1594925-supplementary.pdf]

Table S1. Questionnaire on behavioural risk factors for oral health on Quarantine during Covid-19 pandemic

Ques tion	Parameter	Questions, Response Scale and Romanian Translations (*italic*)	
**Q1**	Oral health self-assessment	How would you describe your current oral health?	*Cum ați descrie starea dumneavoastră de sănătate orală?*
		1. Excellent<br>2. Very good<br>3. Good<br>4. Satisfactory<br>5. Very poor<br>6. I don't know	*1. Excelentă<br>2. Foarte bună<br>3. Bună<br>4. Satisfăcătoare<br>5. Foarte slabă<br>6. Nu știu*
**Q2**	Toothbrushing Frequency	How often did you brush your teeth?<br>a) During lockdown<br>b) Before lockdown	*Cât de des v-ați periat dinții?<br>a) Pe timpul carantinei<br>b) Înainte de carantină*
		1. More than twice a day<br>2. Twice a day<br>3. Once a day<br>4. Several times a week<br>5. Never	*1. Mai mult de 2 ori pe zi<br>2. De 2 ori pe zi<br>3. O dată pe zi<br>4. De câteva ori pe săptămână<br>5. Niciodată*
**Q3**	Toothbrushing Duration	How long was the duration of toothbrushing?<br>a) During lockdown<br>b) Before lockdown	*Cât timp ați acordat periajului dentar?<br>a) Pe timpul carantinei<br>b) Înainte de carantină*
		1. 30 seconds<br>2. 1 minute<br>3. 2 minutes<br>4. More than 2 minutes	*1. 30 secunde<br>2. 1 minut<br>3. 2 minute<br>4. Mai mult de 2 minute*
**Q4**	Toothbrush Type	What type of toothbrush did you use?<br>a) During lockdown<br>b) Before lockdown	*Ce tip de periuță ați folosit?<br>a) Pe timpul carantinei<br>b) Înainte de carantină*
		1. Manual<br>2. Powered<br>3. Both manual and powered	*1. Manuală<br>2. Electrică<br>3. Atât manual cât și electrică*
**Q5**	Use of dental floss	Did you use dental floss?<br>a) During lockdown<br>b) Before lockdown	*Ați utilizat ața dentară?<br>a) Pe timpul carantinei<br>b) Înainte de carantină*
		1. Yes<br>2. No	*1. Da<br>2. Nu*
**Q6**	Frequency of using dental	Did you use daily the dental floss?<br>a) During lockdown<br>b) Before lockdown	*Ați utilizat zilnic ață dentară?<br>a) Pe timpul carantinei<br>b) Înainte de carantinâ*

	floss	1. Yes<br>2. No	*1. Da*<br>*2. Nu*
**Q7**	Use of other secondary oral hygiene products	What other secondary oral hygiene products did you use daily (apart from toothpaste and toothbrush, dental floss)?<br>a) During lockdown<br>b) Before lockdown	*Ce alte produse pentru igienă orală ați utilizat zilnic (în afară de pastă, periuță și ață dentară)?*<br>*a) Pe timpul carantinei*<br>*b) Înainte de carantină*
		1. Mouthwash<br>2. Toothpicks<br>3. Interdental brushes<br>4. Oral irrigator	*1. Apă de gură*<br>*2. Scobitori*<br>*3. Periuțe interdentare*<br>*4. Duș bucal*
**Q8**	Frequency of snacks per day	How many times a day did you eat snacks between the 3 main meals?<br>a) During lockdown<br>b) Before lockdown	*De câte ori pe zi luați gustări între cele 3 mese principale?*<br>*a) Pe timpul carantinei*<br>*b) Înainte de carantină*
		1. Never<br>2. 1-2 times<br>3. 3-4 times<br>4. More than 4 times	*1. Deloc*<br>*2. 1-2 ori*<br>*3. 3-4 ori*<br>*4. Mai mult de 4 ori*
**Q9**	Type of pre-dominant snacks between the main meals	Your snacks between main meals were based mostly on:<br>a) During lockdown<br>b) Before lockdown	*Gustările între mesele principale se bazau predominant pe:*<br>*a) Pe timpul carantinei*<br>*b) Înainte de carantină*
		1. Sweets<br>2. Fruits/vegetables<br>3. Starchy food (biscuits/pretzels)<br>4. Sandwich<br>5. Dairy products (yogurt/kefir)<br>6. Chips/nachos/popcorn)<br>7. Various (a wide-range of food products)	*1. Dulciuri*<br>*2. Fructe/legume*<br>*3. Făinoase (biscuiți/covrigei)*<br>*4. Sandwich*<br>*5. Lactate (iaurt/chefir)*<br>*6. Chipsuri/nachos/popcorn*<br>*7. Variat (o gamă largă de produse)*

Question	Parameter	Questions, Response Scale and Romanian Translations (*italic*)	
**Q10**	Consumption of non-alcoholic beverages	Did you drink beverages?<br>a) During lockdown<br>b) Before lockdown	*Ați consumat sucuri carbogazoase/îndulcite?*<br>*a) Pe timpul carantinei*<br>*b) Înainte de carantină*
		1. Several times a day<br>2. Once a day<br>3. Several times a week<br>4. Seldom<br>5. Never	*1. De câteva ori pe zi*<br>*2. O dată pe zi*<br>*3. De câteva ori pe săptămână*<br>*4. Rareori*<br>*5. Niciodată*
**Q 11**	Smoking habit	Did you smoke?<br>a) During lockdown<br>b) Before lockdown	*Ați fumat?*<br>*a) Pe timpul carantinei*<br>*b) Înainte de carantină*
		1. Never<br>2. Occasional<br>3. 1-10 cigarettes/day<br>4. 11-20 cigarettes/day<br>5. More than 20 cigarettes/day	*1. Niciodată*<br>*2. Ocazional*<br>*3. 1-10 țigări/zi*<br>*4. 11-20 țigări/zi*<br>*5. Mai mult de 20 țigări/zi*
**Q12**	Working/studying place	During the lockdown you worked/studyied:	*Pe durata stării de urgență ați lucrat:*
		1. Not at all<br>2. From home<br>3. At the working place<br>4. Partly from home, partly at the working place	*1. Deloc*<br>*2. De acasă*<br>*3. La locul de muncă*<br>*4. Pațial acasă, parțial la locul de muncă*
**Q13**	Medical/non-medical group	Do you work/study in the medical / dental field?	*Profesați in domeniul medical/stomatologic?*
		1. Yes<br>2. No	*1. Da*<br>*2. Nu*
**Q14**	Location during the lockdown	During the lockdown, you lived:	*Pe durata stării de urgență ați locuit:*
		1. In Romania<br>2. In other country	*1. În România*<br>*2. În altă țară*
**Q15**	Studying field	In which university field do you study (open question restricted to students)	*În ce domeniu studiați (întrebare deschisă adresată doar participanților studenți)*
**Q16**	Age	Age	*Vârsta*
**Q17**	Gender	Sex	*Sexul*
		1. Male<br>2. Female<br>3. I choose not to answer	*1. Masculin*<br>*2. Feminin*<br>*3. Aleg să nu răspund*
